# Mechanisms of FLASH effect

**DOI:** 10.3389/fonc.2022.995612

**Published:** 2022-09-23

**Authors:** Binwei Lin, Dan Huang, Feng Gao, Yiwei Yang, Dai Wu, Yu Zhang, Gang Feng, Tangzhi Dai, Xiaobo Du

**Affiliations:** ^1^ National Health Commission (NHC) Key Laboratory of Nuclear Technology Medical Transformation, Mianyang Central Hospital, Department of Oncology, Mianyang Central Hospital, Mianyang, China; ^2^ State Key Laboratory of Ultrasound in Medicine and Engineering, College of Biomedical Engineering, Chongqing Medical University, Chongqing, China; ^3^ Department of Radiology Mianyang Central Hospital, Mianyang, China; ^4^ Institute of Applied Electronics, China Academy of Engineering Physics, Mianyang, China

**Keywords:** FLASH radiotherapy, conventional dose-rate radiotherapy, mechanism, oxygen, radiobiology

## Abstract

FLASH radiotherapy (FLASH-RT) is a novel radiotherapy technology defined as ultra-high dose rate (≥ 40 Gy/s) radiotherapy. The biological effects of FLASH-RT include two aspects: first, compared with conventional dose rate radiotherapy, FLASH-RT can reduce radiation-induced damage in healthy tissue, and second, FLASH-RT can retain antitumor effectiveness. Current research shows that mechanisms of the biological effects of FLASH-RT are related to oxygen. However, due to the short time of FLASH-RT, evidences related to the mechanisms are indirect, and the exact mechanisms of the biological effects of FLASH-RT are not completely clear and some are even contradictory. This review focuses on the mechanisms of the biological effects of FLASH-RT and proposes future research directions.

## Introduction

Cancer is one of the leading causes of human death (1). Radiotherapy, as one of the main treatments for cancer, can improve the overall survival time (2) and quality of life (3) and can achieve a radical cure (4) in patients with malignancy. However, the overall anti-tumor effect of radiotherapy is still limited (5). The dose limitation of organs at risk surrounding the cancer leads to the relatively insufficient target dose, and this may be the key factor affecting the anti-tumor effect. Modern radiotherapy technologies, such as volumetric modulated arc therapy, tomotherapy, and proton radiotherapy, can optimize the dose distribution (6, 7) and reduce the toxicity in normal tissues; however, the enhancement of anti-tumor effect is limited (8).

FLASH radiotherapy (FLASH-RT) is a novel radiotherapy technology defined as ultra-high dose rate (≥ 40 Gy/s) radiotherapy. Compared with conventional radiotherapy (COVN-RT), FLASH-RT can effectively reduce the toxicity in normal tissues and provide similar anti-tumor effects, which is defined as the FLASH effect (9). Preclinical studies have confirmed that FLASH-RT can effectively reduce the toxicities in lung (10, 11), intestine (12), brain (13), and skin (14), and retain the anti-tumor effect on cancer (11, 15, 16). Due to these encouraging results, FLASH-RT is considered as a revolutionary technology in the field of radiotherapy (17). However, due to the short time of FLASH-RT, evidences regarding the mechanism of FLASH effect are indirect. Therefore, the exact mechanisms of the biological effects of FLASH-RT are not completely clear and some are even contradictory.

The purpose of this review is to provide a relatively brief literature review and discuss the mechanism of the FLASH effect and mainly review the research progress in two aspects: physical-chemical mechanism and biological mechanism.

## Mechanism of FLASH effect

**Figure 1:**
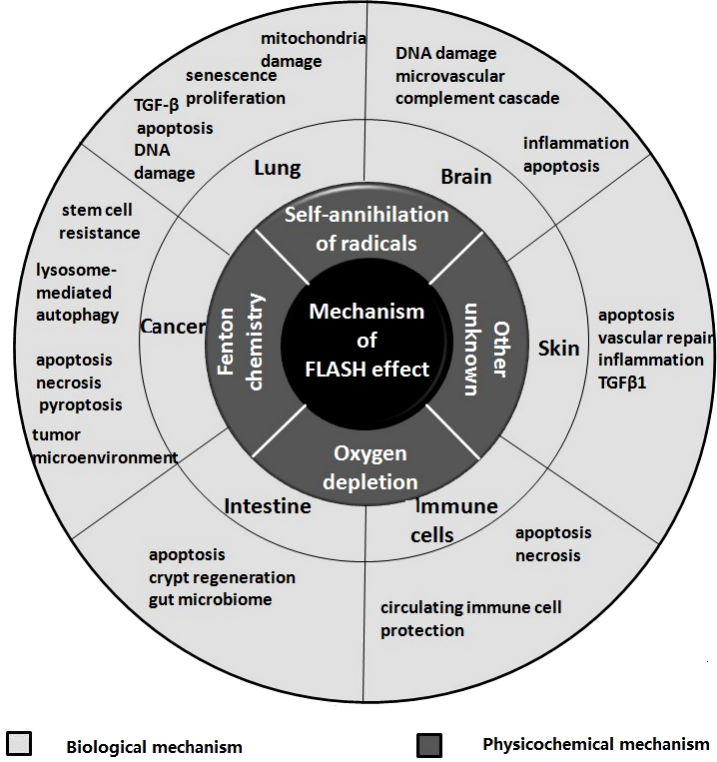
Themechanisms of FLASH effect.

The mechanism of FLASH effect included the physical-chemical mechanism and biological mechanism, the organs involved include lung, brain, intestine, skin etc (**Figure 1** and **Table 1**).

**Table 1:** Summary of published studies on FLASH effect mechanism.

System	Author(s)	Year	Modal	Radiation source	Dose rate(total dose)	Factors relate to Flash effect
Lung	Favaudon V (10)	2014	mice	electron	≥40 Gy/s(17Gy)	TGF-β, Acute apoptosis of vascular endothelial cells
	Fouillade C (18)	2020	mice	electron	≥40 Gy/s(17Gy)	DNA damage, inflammation, proliferation of progenitor, stem cell senescence
	Guo Z (19)	2022	lung fibroblasts	proton	100 Gy/s	mitochondria damage, Drp1-mediated mitochondrial homeostasis
	Buonanno M (20)	2019	lung fibroblasts	proton	1000 Gy/s (>10Gy)	TGFβ, senescence, DNA damage, inflammation
Brain	Montay-Gruel P (21)	2020	mice	electron	5.6×10^6^Gy/s(10Gy)	astrogliosis, complement cascade, inflammation
	Allen BD (22)	2020	mice	electron	2,500 Gy/s (25 Gy) or 5.6×10^6^ Gy/s(10Gy)	apoptosis of neurocyte, microvasculature integrity
	Dokic I (23)	2022	mice	proton	120 Gy/s (10Gy)	DNA damage, preservation of microvascular, reduction of microglia/macrophage regulated associated inflammation
Intestine	Levy K (16)	2020	mice	electron	216 Gy/s (14Gy)	a greater number of regenerating crypts due to less DNA damage and apoptosis of crypt base columnar stem cells
	Kim MM (24)	2022	mice	proton	106.2~118.5Gy/s (15,18Gy)	retain the regenerative capacity of crypt cells
	Ruan JL (25)	2022	mice	electron	2.2-5.9 × 10^6^ Gy/s (7.5~20 Gy)	spare small intestinal crypts and reduce changes in gut microbiome
	Zhu H (26)	2022	mice	X-ray	>150 Gy/s (10Gy,15Gy)	inflammatory blood cells, pro-inflammatory cytokines and lipid peroxidation
Skin	Velalopoulou A (27)	2021	mice	proton	69~124 Gy/s (30Gy, 45Gy)	apoptosis and vascular repairsignal pathways, inflammation, TGFβ1
Immune system	Bozhenko VK (28)	2019	normal lymphocytes, malignant lymphoma cells	photon	1~4×10^9^Gy/min(1~4Gy)	apoptosis, necrosis
	Jin JY (29)	2020	computation study	–	0.0017~333 Gy/s (2~50 Gy)	protect circulating blood cells
Breast cancer	Yang G (30)	2021	breast cancer cell	ion beams	10^9^Gy/s (6~9Gy)	radio-resistance of cancer stem cell may associate with the increase of lysosome-mediated autophagy, and the decrease of apoptosis, necrosis and pyroptosis
Ovarian cancer	Eggold JT (31)	2022	mice	electron	210 Gy/s (14Gy)	regulatory T cells and CD8+ T cells infiltration, tumor microenvironment
Glioblastoma	Ohsawa D (32)	2022	mice	electron	66 Gy/s (8Gy×2, 12.5 Gy×2)	anti-tumor immune function

## Physical-chemical mechanism hypothesis

Free radicals produced by radiation are the root cause of radiation damage. Some studies try to explain the effects of FLASH-RT and COVN-RT by the differences of physical-chemical reaction after radiation.

### Oxygen depletion

Oxygen is considered as an important radiation sensitizer (33). Compared with hypoxic tissue, oxygen rich tissue has stronger radiosensitivity under the same radiation conditions (34). As FLASH-RT can complete the irradiation in a very short time (microseconds), the oxygen in the tissue is rapidly exhausted, and it is too fast to be supplemented by the circulating blood. This results in the relative lack of oxygen in the tissue compared with COVN-RT (irradiation completed in a few minutes), which may be one of the reasons why FLASH-RT can protect the normal tissue (35).The basis for the formation of this hypothesis comes from the early *in vitro* studies on bacteria (36) and mammalian cells (37). It is believed that hypoxia can lead to radiation resistance under ultra-high dose rate irradiation, and this resistance effect reached the maximum under nitrogen condition (oxygen concentration: 0%) (37). Hornsey et al. further supported this hypothesis through *in vivo* experiments. When the dose rate was >6 krad/min, the mortality after whole-body irradiation (in the oxygen inhalation state during irradiation) decreased; however, when the mice were in the nitrogen breathing state during irradiation, the protective effect of ultra-high dose rate radiotherapy was lost (38).

Increasing evidences show that the hypothesis of oxygen depletion cannot fully explain the protective effect of FLASH-RT on normal tissues. Firstly, Jansen et al. measured the change of oxygen content in pure water after FLASH-RT, and found that although FLASH-RT could indeed consume more oxygen than COVN-RT, it could not deplete all oxygen (39). Moreover, when the total dose of FLASH-RT was 10 Gy, oxygen consumption was not detected *in vitro* (39).This contradicted the result that 10 Gy FLASH-RT can better preserve the neural function of mice than COVN-RT in animal experiments (40, 41). Secondly, Epp et al. found that the protective effect of FLASH-RT on mammalian cells occurred when the oxygen concentration is very low (less than 0.5%) (37). Adrian et al. (42) found that FLASH-RT showed a higher cell survival rate than COVN-RT only under hypoxic conditions (oxygen concentration less than 4.4%) and when the total dose exceeded 5-10Gy. One possible explanation is that only when the oxygen concentration is low, FLASH-RT can completely consume the oxygen. However, Gabriel et al. (43) found that the protective effect of FLASH-RT was observed in MDA-MB-231, MCF7 and HeLa cell lines under normoxic conditions (air oxygen concentration). Moreover, the protective effect of FLASH-RT was also observed in oxygen rich tissues such as lung (10). In addition the oxygen depletion hypothesis cannot explain that FLASH-RT and COVN-RT have similar antitumor effects. Because the tumor tissue is relatively hypoxic, FLASH-RT will lead to tumor cell resistance rather than retain the anti-tumor effect.

### Metabolism of peroxidized compounds and Fenton chemistry

Since the oxygen depletion hypothesis cannot fully explain the FLASH effect, Spitz et al. (44) believed that the difference in metabolism of peroxidized compounds and labile iron content between the tumor and normal tissue may be the mechanism of FLASH-RT in reducing normal tissue damage and retaining anti-tumor effect. Compared with tumor tissues, normal tissues retain the metabolic process of peroxidized compounds, therefore, normal tissues have lower peroxidized compounds than tumor tissues, and the labile iron content in normal tissues is lower, which is not conducive to the damage of Fenton chemistry to normal tissues (44). However, Spitz et al. only reasoned from the theoretical model, and subsequent experimental verification, especially the use of animal model of gene knockout of key metabolic enzymes, is very important to verify the Spitz ‘s theory.

### Free radical recombination

Abolfath et al. (45) found that FLASH effect was related to O_2_ concentration by simulating DNA damage models under different conditions. In normal tissues (oxygen concentration 4%-5%), oxygen and DNA molecules form reactive oxygen species (ROS) after radiation. Compared with COVN-RT, the concentration of ROS under FLASH-RT was higher, which lead to the reorganization of ROS free radicals. Subsequently, as the ROS load was reduced, the damage of FLASH-RT to normal tissues was lowered. However, in tumor tissues, the presence of hypoxia (oxygen concentration <0.4%), the production of ROS decreased, resulting in the loss of tissue protection. Labarbe et al. (46) further proposed a theoretical model based on the formation and decay dynamics of ROS. It was found that peroxyl radicals (ROO) was the key component leading to radiation damage, and there was a correlation between the area under the ROO. curve and the probability of normal tissue complications. Compared with COVN-RT, the production rate of ROO. under FLASH-RT was significantly higher. The reduction of ROO. caused by free radical recombination may play a major role in the tissue protection of FLASH-RT. Lai et al. (47) also concluded by using micro-Monte Carlo simulation method that when the dose rate is 10^7^Gy/s and the dose reached 30Gy, it was difficult to exhaust the tissue oxygen with an initial concentration of 0.01%-21%. Compared with COVN-RT, FLASH-RT has higher instantaneous ROO. concentration, and the decrease of ROO. content caused by self-recombination may be the reason of FLASH effect. However, the theory of free radical recombination only comes from model inference. Because the time of free radical recombination is very short, it is difficult to verify it under experimental conditions. Interestingly, Blain et al. (48) found that the production of H_2_O_2_ (one kind of ROS) was significantly reduced in FLASH-RT group when compared with that in COVN-RT group though *in vitro* study. Moreover, the decrease degree of H_2_O_2_ is negatively correlated with the dose rate. When the FLASH dose rate is lower than 100Gy/s, the decrease of H_2_O_2_ is more dramatic. When the FLASH dose rate exceeded 60000Gy/s, the decrease degree of H_2_O_2_ reaches a platform (38% ± 4%). However, H_2_O_2_ cannot represent ROS, future studies are needed to compare the differences of other ROS between FLASH-RT and COVN-RT.

## Biological mechanism

### Effects of FLASH-RT on stem cells

The reduction of stem cell senescence may play an important role in FLASH-RT protection effect. Unlike apoptosis, senescent cells could secrete prion flammatory cytokines, such as IL-6, TGF-β and IL-1α, and lead to subsequent pulmonary fibrosis (18). Moreover, the stem cell senescence hinders the process of cell regeneration after radiation injury (18, 49). Fouillade et al. (49) conducted a preclinical study to evaluate the role of stem cell senescence in the protection of normal tissues by FLASH-RT. C57BL/6J mice were irradiated under FLASH-RT (>40Gy/s, 17Gy) and COVN-RT (<0.003Gy/s, 17 Gy) conditions using electron. They found that compared with COVN-RT group, FLASH-RT had less lung injury and similar anti-tumor effect. Further studies showed that the lung protective effect of FLASH-RT may be related to the retention of stem cell replication ability, because they found that the number of senescence stem cells (reduced or disappeared replication ability) in the FLASH-RT group decreased by 50% compared with the COVN-RT group. More importantly, Fouillade et al. compared the lung injury between TERC-/- mice (mice with extremely short telomeres, simulating the state of stem cells senescence) and wild mice, the phenomenon of FLASH-RT on lung protection disappeared in TERC-/- mice. Yang et al. (30) compared the damage ability of FLASH-RT to tumor stem cells and normal tumor cells *in vitro*. They concluded that under the condition of FLASH-RT (10^9^Gy/s, 6~9Gy), tumor stem cells and normal tumor cells both will undergo apoptosis, scorch, and necrosis after irradiation. However, tumor stem cells have stronger radiation resistance than normal tumor cells. The radiation resistance of cancer stem cells may be related to the increase of lysosome mediated autophagy. Due to Yang et al. (30) did not compare the damage of FLASH-RT and COVN-RT on tumor stem cells, whether tumor stem cells affect the retention anti-tumor effect of FLASH-RT needs further study.

The retention of the stem cell division ability by FLASH-RT may only partially explain the protective effect of FLASH-RT on normal tissues, and there may be other relevant mechanisms for the retention of the anti-tumor effect (27). Simultaneously, more studies are needed to verify the experimental results of Fouillade et al.

### Effect of FLASH-RT on immune function

Radiation damage has been shown to be a sterile inflammatory process (50), and immune function plays an important role in radiation injury (51). TGF-β has been proved to be an inflammatory factor that participates in the process of DNA damage, repair and cellular inflammatory response and promotes the formation of radiation-induced pulmonary fibrosis (52). Several studies show that the expression of TGF-β in FLASH-RT group was significantly decreased when compared with COVN-RT group (10, 20, 27). Fouillade et al (49) studied the role of immune inflammatory change in lung injury after FLASH-RT. The animal model and radiotherapy parameters used in the study were consistent as previously described. The results showed that FLASH-RT had less expression of pro-inflammatory factor gene (EGR1) and lower up-regulation of inflammatory factor (TGF-β1, NF-KB) than COVN-RT. Zhu et al. (26) reported the changes of immune and inflammatory responses after FLASH-RT(>150Gy/s, 10Gy and 15Gy) irradiation on the intestine of mice(BALB/c). X-ray was used in Zhu’s study. They found that compared with COVN-RT, the mice in FLASH-RT group had lower intestinal toxicity, inflammatory blood cells (leukocytes, lymphomas, neutrophils), pro-inflammatory cytokines (TNF- α, IL-6, IL-10) and lipid peroxidation were significantly reduced. Preclinical studies suggest that the increase of chronic neuritis associated with microglia activation may be related to radiation-induced brain injury (53, 54). Montay-Gruel et al. (21) studied the brain damage of mice (C57BL/6J) caused by FLASH-RT(5.6 × 10^6^Gy/s, 10Gy) and COVN-RT(0.1Gy/s, 10Gy) using electron beam. They found that the expression level of the markers (GFAP, TLR4) that activate astrocyte proliferation in the brain of FLASH-RT group were significantly lower than that in COVN-RT group. Recently, the experimental results of Dokic et al. (23) also supported that FLASH-RT reduced microglia/macrophage regulated inflammation compared with COVN-RT.

Circulating immune cells may have an important impact on the repair of normal tissues after radiotherapy (55) and the anti-tumor effect (56, 57). Therefore, the protection of circulating immune cells by FLASH-RT may be part of the mechanism of FLASH effect. Jin et al. (29) used computer simulation to evaluate the effects of FLASH-RT and COVN-RT on circulating immune cells. They found that the killing rate of FLASH-RT on circulating immune cells was significantly lower than that of COVN-RT (5%-10% *vs* 90%-100%). However, it should be noted that this research was only computer simulation, and the research results needed to be verified by experiments. Moreover, only the circulating immune cells were considered in this study, rather than the evaluation of immune cells in immune organs and tumor tissues. Whether FLASH-RT can protect the whole immune function needs to be studied in the future. Eggold et al. (31) evaluated the effect of FLASH-RT on immune cells in tumor by establishing an animal model of peritoneal ovarian cancer. It was found that regulatory T cells decreased and CD8^+^ T cells increased in tumors treated with FLASH-RT(210 Gy/s,14Gy) and COVN-RT(0.126 Gy/s,14Gy). When compared with COVN-RT, FLASH-RT group had significantly more T cell infiltration, especially CD8^+^ T. When radiotherapy was combined with PD-1 inhibitor, the anti-tumor effect of FLASH-RT group was better than that of COVN-RT group. The reliability of the results of Eggold’s study needs to be confirmed by more preclinical studies, however, this study suggested that FLASH-RT combined with immunotherapy may have bright prospects.

Immune function may also play an important role in preserving the antitumor effect of FLASH-RT. Recently, Liljedahl et al. (32) used tumor bearing mice to compare the anti-tumor effect of FLASH-RT(66 Gy/s, 8Gy×2 fractions and 12.5 Gy×2 fractions) and COVN-RT(0.133Gy/s, 8Gy×2 fractions and 12.5 Gy×2 fractions), and re-challenged the cured mice after radiotherapy to evaluate the long-term anti-tumor effect. They found that FLASH-RT and COVN-RT had similar anti-tumor effect (median survival time: 100 days *vs* 100 days, p>0.05). Cured mice (FLASH-RT: 8 mice; COVN-RT: 6 mice) were then rechallenged with tumor. The results showed that tumor re-growth was not detected in the additional 100 days.

### Effect of FLASH-RT on blood vessels

Vascular injury caused by radiotherapy is considered to be an important part of radiation injury (58, 59). Favaudon et al. (10) found that FLASH-RT can reduce the acute apoptosis of bronchial vessels compared with COVN-RT. Two studies focus on brain injury showed that FLASH-RT was superior to COVN-RT in protecting the integrity of microvessels in the brain, which may be conducive to the preservation of cognitive function by FLASH-RT (22, 23). However, the current research evidence only supports that FLASH-RT has less vascular damage than COVN-RT, and the impact of FLASH-RT on the upstream gene regulatory pathway is not clear.

### Other possible biological mechanisms

Three preclinical studies show that the protective effect of FLASH-RT on intestinal tract may be related to the protection of intestinal crypt cells by FLASH-RT (16, 24, 25). Ruan et al. (25) also found that FLASH-RT has less impact on intestinal flora than COVN-RT, which may be more conducive to the protection of intestinal function. Guo et al. (19) found that FLASH-RT can reduce mitochondrial damage mediated by Dynamin-1-like protein. Jay et al. (60) believed that FLASH-RT may produce an early transient strong acidic environment, which may be one of the mechanisms of FLASH-RT to protect normal tissues. Ohsawa et al. (61) studied the effect of proton FLASH-RT (40 Gy/s) and COVN-RT (0.05 Gy/s) on DNA damage. They found that compared with COVN-RT, the single strand DNA breakage in FLASH-RT group was significantly reduced, but the double strand DNA breakage was similar. Ohsawa et al. (61) speculated that the FLASH-RT might effectively reduce non lethal damage, such as cell senescence, genomic instability and cell transformation.

## Conclusions

The mechanism of FLASH effect include physical-chemical mechanism, biological mechanism and others, which involved oxygen depletion, Fenton effect, free radical recombination, stem cells, immune function, blood vessels, etc. But the published results on the mechanism of FLASH effect can not fully explain the FLASH effect. More studies are needed to clarify the real mechanisms of FLASH-RT and the weight values of different mechanisms.

## Author contributions

BL and DH draft the manuscript, FG, YY, DW, YZ, GF, and TD participated in the data review and collection for the study. XD conceived of the study, and participated in its design and coordination. All authors contributed to the article and approved the submitted version.

## Funding

This work was financially supported by the NHC Key Laboratory of Nuclear Technology Medical Transformation (Mianyang Central Hospital) (grantnos. 2022HYX008) and Natural Science Foundation of Sichuan Province (grant no.2022NSFSC0849).

## Conflict of interest

The authors declare that the research was conducted in the absence of any commercial or financial relationships that could be construed as a potential conflict of interest.

## Publisher’s note

All claims expressed in this article are solely those of the authors and do not necessarily represent those of their affiliated organizations, or those of the publisher, the editors and the reviewers. Any product that may be evaluated in this article, or claim that may be made by its manufacturer, is not guaranteed or endorsed by the publisher.

## References

[1] Ferlay J Colombet M Soerjomataram I Parkin DM Pineros M Znaor A. Cancer statistics for the year 2020: An overview. Int J Cancer (2021) 149:778–89. doi: 10.1002/ijc.33588 33818764

[2] Cozzi S Alì E Bardoscia L. Stereotactic body radiation therapy (SBRT) for Oligorecurrent/OligoprogressiveMediastinal and hilar lymph node metastasis: A systematic review. Cancers (Basel) (2022) 14(11):2680. doi: 10.3390/cancers14112680 35681659 PMC9179886

[3] Rim CH Park S Yoon WS Shin IS Park HC. Radiotherapy for bone metastases of hepatocellular carcinoma: a hybrid systematic review with meta-analyses. Int J Radiat Biol (2022):1–12. doi: 10.1080/09553002.2022.2094020 35758976

[4] Jicman Stan D Niculet E Lungu M Onisor C Rebegea L Vesa D. Nasopharyngeal carcinoma: A new synthesis of literature data (Review). ExpTher Med (2022) 23(2):136. doi: 10.3892/etm.2021.11059 PMC8756428 35069817

[5] Delaney G Jacob S Featherstone C Barton M. The role of radiotherapy in cancer treatment: estimating optimal utilization from a review of evidence-based clinical guidelines. Cancer (2005) 104(6):1129–37. doi: 10.1002/cncr.21324 16080176

[6] Cheng YK Kuo SH Yen HH Wu JH Chen YC Huang MY. The prognostic significance of pretreatment squamous cell carcinoma antigen levels in cervical cancer patients treated by concurrent chemoradiation therapy and a comparison of dosimetric outcomes and clinical toxicities between tomotherapy and volumetric modulated arc therapy. Radiat Oncol (2022) 17(1):91. doi: 10.1186/s13014-022-02063-w 35549962 PMC9097430

[7] Paganetti H Botas P Sharp GC Winey B. Adaptive proton therapy. Phys Med Biol (2021) 66(22):TR01. doi: 10.1088/1361-6560/ac344f PMC8628198 34710858

[8] Lin SH Hobbs BP Verma V Tidwell RS Smith GL Lei X. Randomized phase IIB trial of proton beam therapy versus intensity-modulated radiation therapy for locally advanced esophageal cancer. J Clin Oncol (2020) 38(14):1569–79. doi: 10.1200/JCO.19.02503 PMC7213588 32160096

[9] Favaudon V Labarbe R Limoli CL. Model studies of the role of oxygen in the FLASH effect. Med Phys (2022) 49(3):2068–81. doi: 10.1002/mp.15129 PMC8854455 34407219

[10] Favaudon V Caplier L Monceau V Pouzoulet F Sayarath M Fouillade C. Ultrahigh dose-rate flash irradiation increases the DifferentialResponse between normal and tumor tissue in mice. Sci Trans Med (2014) 6(245):245ra93–245ra93. doi: 10.1126/scitranslmed.3008973 25031268

[11] Gao F Yang Y Zhu H Wang J Xiao D Zhou Z. First demonstration of the FLASH effect with ultrahigh dose rate high-energy X-rays. Radiother Oncol (2022) 166:44–50. doi: 10.1016/j.radonc.2021.11.004 34774651

[12] Loo BW Schuler E Lartey FM Rafat M King GJ Trovati S. (P003)Delivery of ultra-rapid flash radiation therapy and demonstration ofNormal tissue sparing after abdominal irradiation of mice. Int J Radiat Oncol (2017) 98:E16. doi: 10.1016/j.ijrobp.2017.02.101

[13] Simmons DA Lartey FM Schüler E Rafat M King G Kim A. Reduced cognitive deficits after FLASH irradiation ofWholeMouse brain are AssociatedWith less hippocampal dendritic spine loss and neuroinflammation. Radiother Oncol (2019) 139:4–10. doi: 10.1016/j.radonc.2019.06.006 31253467

[14] Singers Sørensen B Krzysztof Sitarz M Ankjærgaard C Johansen J Andersen CE Kanouta E. *In vivo* validation and tissue sparing factor for acute damage of pencil beam scanning proton FLASH. Radiother Oncol (2022) 167:109–15. doi: 10.1016/j.radonc.2021.12.022 34953933

[15] Montay-Gruel P Acharya MM Gonçalves Jorge P Petit B Petridis IG Fuchs P. Hypo-fractionated FLASH-RT as an effective treatment against glioblastoma that reduces neurocognitive side effects in mice. Clin Cancer Res (2020) 27(3):775–84. doi: 10.1158/1078-0432.CCR-20-0894 PMC7854480 33060122

[16] Levy K Natarajan S Wang J Chow S Eggold JT Loo PE. Abdominal FLASH irradiation reduces radiation-induced gastrointestinal toxicity for the treatment of ovarian cancer in mice. Sci Rep (2020) 10(1):21600. doi: 10.1038/s41598-020-78017-7 33303827 PMC7728763

[17] Maxim PG Keall P Cai J. FLASH radiotherapy: Newsflash or flash in the pan? Med Phys (2019) 46(10):4287–90. doi: 10.1002/mp.13685 31246281

[18] Citrin DE Shankavaram U Horton JA Shield W 3rd Zhao S Asano H. Roleof type II pneumocyte senescence in radiation-induced lung fibrosis. J Natl Cancer Inst (2013) 105:1474–84. doi: 10.1093/jnci/djt212 PMC3787909 24052614

[19] Guo Z Buonanno M Harken A Zhou G Hei TK. Mitochondrial damage response and fate of normal cells exposed to FLASH irradiation with protons. Radiat Res (2022) 197(6):569–82. doi: 10.1667/RADE-21-00181.1 PMC9241019 35290449

[20] Buonanno M Grilj V Brenner DJ. Biological effects in normal cells exposed to FLASH dose rate protons. Radiother Oncol (2019) 139:51–5. doi: 10.1016/j.radonc.2019.02.009 PMC6728238 30850209

[21] Montay-Gruel P Markarian M Allen BD Baddour JD Giedzinski E Jorge PG. Ultra-High-Dose-Rate FLASH irradiation limits reactive gliosis in the brain. Radiat Res (2020) 194(6):636–45. doi: 10.1667/RADE-20-00067.1 PMC7856066 32853387

[22] Allen BD Acharya MM Montay-Gruel P Jorge PG Bailat C Petit B. Maintenance of tight junction integrity in the absence of vascular dilation in the brain of mice exposed to ultra-High-Dose-Rate FLASH irradiation. Radiat Res (2020) 194(6):625–35. doi: 10.1667/RADE-20-00060.1 PMC7773228 33348373

[23] Dokic I Meister S Bojcevski J Tessonnier T Walsh D Knoll M. Neuroprotective effects of ultra-high dose rate FLASH Bragg peak proton irradiation. Int J Radiat Oncol Biol Phys (2022) 113(3):614–23. doi: 10.1016/j.ijrobp.2022.02.020 PMC11034835 35196536

[24] Kim MM Verginadis II Goia D Haertter A Shoniyozov K Zou W. Comparison of FLASH proton entrance and the spread-out Bragg peak dose regions in the sparing of mouse intestinal crypts and in a pancreatic tumor model. Cancers (Basel) (2021) 13(16):4244. doi: 10.3390/cancers13164244 34439398 PMC8392865

[25] Ruan JL Lee C Wouters S Tullis IDC Verslegers M Mysara M. Irradiation at ultra-high (FLASH) dose rates reduces acute normal tissue toxicity in the mouse gastrointestinal system. Int J Radiat Oncol Biol Phys (2021) 111(5):1250–61. doi: 10.1016/j.ijrobp.2021.08.004 PMC7612009 34400268

[26] Zhu H Xie D Yang Y Huang S Gao X Peng Y. Radioprotective effect of X-ray abdominal FLASH irradiation: Adaptation to oxidative damage and inflammatory response may be benefiting factors. Med Phys (2022) 49(7):4812–22. doi: 10.1002/mp.15680 35451077

[27] Velalopoulou A Karagounis IV Cramer GM Kim MM Skoufos G Goia D. FLASH proton radiotherapy spares normal epithelial and mesenchymal tissues while preserving sarcoma response. Cancer Res (2021) 81(18):4808–21. doi: 10.1158/0008-5472.CAN-21-1500 PMC8715480 34321243

[28] Bozhenko VK Ivanov AV Kulinich TM Smirnov VP Shishkin AM Solodky VA. Comparison of biological effects of ?-radiation of low and ultra-high dose rate on lymphocytes and cultured human malignant lymphoma cells. Bull Exp Biol Med (2019) 166(6):785–7. doi: 10.1007/s10517-019-04440-0 31028581

[29] Jin JY Gu A Wang W Oleinick NL Machtay M Spring Kong FM. Ultra-high dose rate effect on circulating immune cells: A potential mechanism for FLASH effect? Radiother Oncol (2020) 149:55–62. doi: 10.1016/j.radonc.2020.04.054 PMC7442672 32387486

[30] Yang G Lu C Mei Z Sun X Han J Qian J. Association of cancer stem cell radio-resistance under ultra-high dose rate FLASH irradiation with lysosome-mediated autophagy. Front Cell Dev Biol (2021) 9:672693. doi: 10.3389/fcell.2021.672693 33996830 PMC8116574

[31] Eggold JT Chow S Melemenidis S Wang J Natarajan S Loo PE. Abdominopelvic FLASH irradiation improves PD-1 immune checkpoint inhibition in preclinical models of ovarian cancer. Mol Cancer Ther (2022) 21(2):371–81. doi: 10.1158/1535-7163.MCT-21-0358 PMC9229218 34866044

[32] Liljedahl E Konradsson E Gustafsson E Jonsson KF Olofsson JK Ceberg C. Long-term anti-tumor effects following both conventional radiotherapy and FLASH in fully immunocompetent animals with glioblastoma. Sci Rep (2022) 12(1):12285. doi: 10.1038/s41598-022-16612-6 35853933 PMC9296533

[33] Read J. Mode of action of X-raydoses given with different oxygenconcentrations. Br J Radiol (1952) 25:336–8. doi: 10.1259/0007-1285-25-294-336 14925316

[34] Spiro IJ Ling CC Stickler R Gaskill J. Oxygen radiosensitisation at low dose rate. Br J Radiol. (1985) 58(688):357–63. doi: 10.1259/0007-1285-58-688-357 4063678

[35] Weiss H Epp ER Heslin JM Ling CC Santomasso A. Oxygen depletion in cells irradiated at ultra-high dose-rates and at conventional dose-rates. Int J Radiat Biol Relat Stud Phys Chem Med (1974) 26(1):17–29. doi: 10.1080/09553007414550901 4607987

[36] Dewey DL Boag JW. Modification of the oxygen EffectWhen bacteria are GivenLarge pulses of radiation. Nature (1959) 183(4673):1450. doi: 10.1038/1831450a0 13657161

[37] Epp ER Weiss H Djordjevic B Santomasso A. The radiosensitivity of cultured mammalian cells exposed to single high intensity pulses of electrons in various concentrations of oxygen. Radiat Res (1972) 52(2):324–32. doi: 10.2307/3573572 4566064

[38] Hornsey S Bewley DK. Hypoxia in mouse intestine induced by electron irradiation at high dose-rates. Int J Radiat Biol Relat Stud Phys Chem Med (1971) 19(5):479–83. doi: 10.1080/09553007114550611 5314348

[39] Jansen J Knoll J Beyreuther E Pawelke J Skuza R Hanley R. Does FLASH deplete oxygen? experimental evaluation for photons, protons, and carbon ions. Med Phys (2021) 48(7):3982–90. doi: 10.1002/mp.14917 33948958

[40] Montay-Gruel P Acharya MM Petersson K Alikhani L Yakkala C Allen BD. Long-term neurocognitive benefits of FLASH radiotherapy driven by reduced reactive oxygen species. Proc Natl Acad Sci USA (2020) 117(41):25946–7. doi: 10.1073/pnas.1901777116 PMC6561167 31097580

[41] Montay-Gruel P Bouchet A Jaccard M Patin D Serduc R Aim W. X-Rays can trigger the FLASH effect: Ultra-high dose-rate synchrotron light source prevents normal brain injury after whole brain irradiation in mice. Radiother Oncol (2018) 129(3):582–8. doi: 10.1016/j.radonc.2018.08.016 30177374

[42] Adrian G Konradsson E Lempart M Bäck S Ceberg C Petersson K. The FLASH effect depends on oxygen concentration. Br J Radiol (2020) 93(1106):20190702. doi: 10.1259/bjr.20190702 31825653 PMC7055454

[43] Adrian G Konradsson E Beyer S Wittrup A Butterworth KT McMahon SJ. Cancer cells can exhibit a sparing FLASH effect at low doses under normoxic *In vitro*-conditions. Front Oncol (2021) 11:686142. doi: 10.3389/fonc.2021.686142 34395253 PMC8358772

[44] Spitz DR Buettner GR Petronek MS St-Aubin JJ Flynn RT Waldron TJ. An integrated physico-chemical approach for explaining the differential impact of FLASH versus conventional dose rate irradiation on cancer and normal tissue responses. Radiother Oncol (2019) 139:23–7. doi: 10.1016/j.radonc.2019.03.028 PMC6761031 31010709

[45] Abolfath R Grosshans D Mohan R. Oxygen depletion in FLASH ultra-high-dose-rate radiotherapy: A molecular dynamics simulation. Med Phys (2020) 47(12):6551–61. doi: 10.1002/mp.14548 33089504

[46] Labarbe R Hotoiu L Barbier J Favaudon V. A physicochemical model of reaction kinetics supports peroxyl radical recombination as the main determinant of the FLASH effect. Radiother Oncol (2020) 153:303–10. doi: 10.1016/j.radonc.2020.06.001 32534957

[47] Lai Y Jia X Chi Y. Modeling the effect of oxygen on the chemical stage of water radiolysis using GPU-based microscopic Monte Carlo simulations, with an application in FLASH radiotherapy. Phys Med Biol (2021) 66(2):025004. doi: 10.1088/1361-6560/abc93b 33171449 PMC8236313

[48] Blain G Vandenborre J Villoing D Fiegel V Fois GR Haddad F. Proton irradiations at ultra-high dose rate vs. conventional dose rate: Strong impact on hydrogen peroxide yield. Radiat Res (2022). doi: 10.1667/RADE-22-00021.1 35675499

[49] Fouillade C Curras-Alonso S Giuranno L Quelennec E Heinrich S Bonnet-Boissinot S. FLASH irradiation spares lung progenitor cells and limits the incidence of radio-induced senescence. Clin Cancer Res (2020) 26(6):1497–506. doi: 10.1158/1078-0432.CCR-19-1440 31796518

[50] Yahyapour R Amini P Rezapour S Cheki M Rezaeyan A Farhood B. Radiation-induced inflammation and autoimmune diseases. Mil Med Res (2018) 5(1):9. doi: 10.1186/s40779-018-0156-7 29554942 PMC5859747

[51] Najafi M Motevaseli E Shirazi A Geraily G Rezaeyan A Norouzi F. Mechanisms of inflammatory responses to radiation and normal tissues toxicity: clinical implications. Int J Radiat Biol (2018) 94(4):335–56. doi: 10.1080/09553002.2018.1440092 29504497

[52] Wang J Xu Z Wang Z Du G Lun L. TGF-beta signaling in cancer radiotherapy. Cytokine (2021) 148:155709. doi: 10.1016/j.cyto.2021.155709 34597918

[53] Acharya MM Baulch JE Lusardi TA Allen BD Chmielewski NN Baddour AA. Adenosine kinase inhibition protects against cranial radiation-induced cognitive dysfunction. Front Mol Neurosci (2016) 9:42. doi: 10.3389/fnmol.2016.00042 27375429 PMC4891332

[54] Zhou H Liu Z Liu J Wang J Zhou D Zhao Z. Fractionated radiation-induced acute encephalopathy in a young rat model: cognitive dysfunction and histologic findings. AJNR Am J Neuroradiol (2011) 32:1795–800. doi: 10.3174/ajnr.A2643 PMC7966005 21920857

[55] Wirsdörfer F Jendrossek V. The role of lymphocytes in radiotherapy-induced adverse late effects in the lung. Front Immunol (2016) 1214:7:591. doi: 10.3389/fimmu.2016.00591 PMC5155013 28018357

[56] Campian JL Ye X Brock M Grossman SA. Treatment-related lymphopenia in patients with stage III non-small-cell lung cancer. Cancer Invest (2013) 31:183–8. doi: 10.3109/07357907.2013.767342 PMC4596242 23432821

[57] Davuluri R Jiang W Fang P Xu C Komaki R Gomez DR. Lymphocyte nadir and esophageal cancer survival outcomes after chemoradiation therapy. Int J Radiat Oncol Biol Phys (2017) 99(1):128–35. doi: 10.1016/j.ijrobp.2017.05.037 28816138

[58] Fauquette W Amourette C Dehouck MP Diserbo M. Radiation-induced blood-brain barrier damages: An *in vitro* study. Brain Res (2012) 1433:114–26. doi: 10.1016/j.brainres.2011.11.022 22153623

[59] Yuan H Gaber MW McColgan T Naimark MD Kiani MF Merchant TE. Radiation-induced permeability and leukocyte adhesion in the rat blood-brain barrier: modulation with anti-ICAM-1 antibodies. Brain Res (2003) 969:59–69. doi: 10.1016/S0006-8993(03)02278-9 12676365

[60] Jay-Gerin JP. Ultra-high dose-rate (FLASH) radiotherapy: Generation of early, transient, strongly acidic spikes in the irradiated tumor environment. Cancer Radiother (2020) 24(4):332–4. doi: 10.1016/j.canrad.2019.11.004 32446537

[61] Ohsawa D Hiroyama Y Kobayashi A Kusumoto T Kitamura H Hojo S. DNA Strand break induction of aqueous plasmid DNA exposed to 30 MeV protons at ultra-high dose rate. J Radiat Res (2022) 63(2):255–60. doi: 10.1093/jrr/rrab114 PMC8944314 34952540

